# Altered cardiac muscle mTOR regulation during the progression of cancer cachexia in the *Apc^Min/+^* mouse

**DOI:** 10.3892/ijo.2013.1893

**Published:** 2013-04-11

**Authors:** NANDINI D.P.K. MANNE, M. LIMA, R.T. ENOS, P. WEHNER, J.A. CARSON, ERIC BLOUGH

**Affiliations:** 1 Center for Diagnostic Nanosystems, Marshall University, Huntington, WV;; 2 Departments of Pharmacology, Physiology and Toxicology, Joan C. Edwards School of Medicine, Marshall University, Huntington, WV;; 3 Cardiology, Joan C. Edwards School of Medicine, Marshall University, Huntington, WV;; 4 Department of Pharmaceutical Science and Research, School of Pharmacy, Marshall University, Huntington, WV;; 5 Integrated Muscle Biology Laboratory, Applied Physiology Division Exercise Science, University of South Carolina, SC, USA

**Keywords:** cachexia, *Apc*^*Min/+*^, heart, Akt, AMPK, mTOR

## Abstract

Cancer cachexia is a muscle wasting condition that occurs in response to a malignant growth in the body. The mechanisms regulating cardiac muscle mass with cachexia are not well understood. Using the *Apc^Min/+^* mouse model of colorectal cancer, we investigated how cachexia affects the regulation of 5′-adenosine monophosphate-activated protein kinase (AMPK), protein kinase B (Akt) and mammalian target of rapamycin (mTOR) signaling in the heart. Compared to age-matched C57BL/6 (BL6) mice, *Apc^Min/+^* body mass and heart mass were lower at 12 (11±5 and 8±3%, respectively) and 20 weeks (26±3 and 6±4%, respectively) of age (P<0.05). Diminished heart mass in the 20-week-old *Apc^Min/+^* mice coincided with a decreased rate of myofibrillar protein synthesis and increased AMPKα phosphorylation. Cachexia decreased mTOR phosphorylation and the phosphorylation of the mTOR substrates, S6 ribosomal protein and 4EBP1 independent of Akt activation. These changes in mTOR-related protein signaling were accompanied by modest increases in the amount of Beclin1 but not protein ubiquitination or cardiomyocyte apoptosis. Taken together, these data suggest that loss of cardiac mass during cachexia progression in the *Apc^Min/+^* mouse is associated with an Akt-independent suppression of anabolic signaling and evidence of increased autophagy.

## Introduction

Colorectal cancer is most commonly associated with a mutation in adenomatous polyposis coli (APC) gene and is the second leading cause of deaths due to cancer in the United States (1). The *Apc^Min/+^* mouse exhibits a nonsense mutation at codon 850 in the APC gene (2) that, similar to that seen in clinical studies, exhibits intestinal neoplasms and develops a cachectic state characterized by severe muscle wasting with increasing tumor burden (3,4). The etiology of cachexia is multifactorial and is most likely mediated, at least in part, by humoral factors that are secreted from or induced by the tumor which cause an imbalance between the rates of protein synthesis and breakdown (5).

The factors which regulate protein turnover in striated muscle have not been fully elucidated, however, studies have suggested that the 5′-adenosine monophosphate-activated protein kinase (AMPK) and PI3K/Akt/mTOR signaling pathways may play key roles (6). AMPK is a key cellular energy sensor that is activated by a diminished intracellular ATP/ADP ratio. Activation of AMPK serves to increase energy availability and suppresses energy demanding processes in the cell. Targets of AMPK activation include the phosphorylation of Unc-51-like kinase-1 (ULK1) which can lead to the activation of autophagy (7). AMPK also suppresses the mammalian target of rapamycin complex 1 (mTORC1) formation, which functions as central regulator of cell growth that when phosphorylated acts to stimulate increases in protein synthesis (8).

While most published reports have focused on the effects of cachexia on skeletal muscle, Burch and colleagues, were amongst the first to note that cachectic cancer patients exhibited evidence of diminished cardiac mass (9). How cancer cachexia may affect AMPK and PI3K/Akt/mTOR signaling in the heart has, to our knowledge, not been reported. We hypothesized that hearts from cachectic *Apc^Min/+^* mice would weigh less than that observed in age-matched control animals and that this response would be associated with decreased anabolic signaling related to the phosphorylation (activation) of AMPK and PI3K/Akt/mTOR signaling. Taken together, our data suggest that loss of cardiac mass during the progression of cachexia in the *Apc^Min/+^* mouse is associated with a diminished rate of protein synthesis that is characterized by suppressed mTOR activity and increases in AMPK phosphorylation.

## Materials and methods

### Animals

All procedures were performed as outlined in the Guide for the Animal Use Review Board at the University of South Carolina. Young (12-week-old, n=6) and adult (20-week-old, n=6) male C57BL/6 and *Apc^Min/+^* mice were originally purchased from Jackson Laboratories and bred at the University of South Carolina’s Animal Resource Facility as previously described (10). Animals were housed under a 12/12-h dark-light cycle at 22±2°C. Mice were fed with standard rodent chow (Harlan Teklad Rodent Diet, #8604, Madison, WI) and water was provided *ad libitum*. After acclimatization the mice were sacrificed according to the regulatory guidelines of University of South Carolina and the samples processed for further analysis.

### Materials

Anti-Akt (#9272), mTOR (#2972), S6 ribosomal protein (#2217), AMPK-α (#2532), Beclin1 (#3738), phosphorylated Akt Thr308 (#9275), phosphorylated Akt Ser473 (#9271), phosphorylated mTOR Ser2448 (#2971), phosphorylated S6 ribosomal protein Ser235/236 (#4858), phosphorylated AMPK-α Thr172 (#2535), phosphorylated Bad (#9295), mouse IgG and rabbit IgG antibodies were purchased from Cell Signaling Technology (Beverly, MA). HeLa whole cell lysate (sc-2200) and L6 + IGF lysate (sc-24727) were purchased from Santa Cruz Biotechnology (Santa Cruz, CA). Enhanced chemiluminescence (ECL) western blotting detection reagent was purchased from Amersham Biosciences (Piscataway, NJ). Restore western blot stripping buffer was obtained from Pierce (Rockford, IL). All other chemicals were purchased from Fisher Scientific (Hanover, IL) or Sigma Aldrich (St. Louis, MO).

### Preparation of protein samples and immunoblotting

Hearts were pulverized using a mortar and pestle in liquid nitrogen until a fine pellet was obtained. Samples were lysed on ice for 15 min in T-PER buffer (2 ml/g tissue weight; Pierce, Rockford, IL) containing protease and phosphatase inhibitors to prevent protein degradation followed by centrifugation for 10 min at 2000 × g to remove particulate matter. This process was repeated twice and the supernatants were collected for the determination of protein concentration using the 660-nm assay (Pierce). Samples were diluted to a final concentration of 2.5 *μ*g/*μ*l in SDS loading buffer, boiled for 5 min at 95°C and 30 *μ*g of protein were separated using 10 or 15% SDS-PAGE gels. Protein detection subsequent to transfer to nitrocellulose membranes were performed as outlined by the antibody manufacturer and visualized using ECL (Amersham Biosciences). Time of exposure at all times was adjusted accordingly to keep the integrated optical densities within a linear and non-saturated range. The intensity of band signal was quantified by imaging software (AlphaEase, FC). GAPDH was used to determine equal loading of protein between lanes and membranes. For direct comparisons between the concentration levels of different signaling molecules, membranes were stripped and re-probed using Restore western blot stripping buffer as detailed by the manufacturer (Pierce).

### Myofibrillar protein synthesis

The rate of heart myofibrillar protein synthesis in an additional set of 20-week-old male C57BL/6 and *Apc^Min/+^* mice (n=6–7) was measured as previously described for skeletal muscle with the following modifications (11,12). Thirty minutes prior to sacrifice, all mice received an intraperitoneal injection of 150 mM ^2^H_5_-phenylalanine (Cambridge Isotopes, MA) in a 75-mM NaCl solution at a dose of 0.02 ml/g body weight. After anesthesia (ketaminexylazine-acepromazine cocktail; 1.4 ml/kg body weight s.c.), whole hearts were excised, rinsed in PBS, quickly weighed, snap frozen in liquid nitrogen and stored at −80°C until further analysis. Hearts were homogenized in ice-cold homogenizing buffer (HB: 50 mM KPO_4_, pH 7.0, 0.25 M sucrose, 1% Triton X-100) and myofibrillar proteins were pelleted by centrifugation. After collection of the supernatants containing free amino acids, myofibrillar proteins were acid hydrolysed and the ratio of ^2^H_5_-phenylalanine to naturally occurring phenylalanine in the myofibrillar proteins and amino acid pool was determined by tandem mass spectrometry (ultra-pressure liquid chromatography-MS/MS), ESI positive mode, by monitoring ions m/z 125 and 120, respectively. Percentage protein synthesis/day was calculated by dividing the ratio of ^2^H_5_-phenylalanine/phenylalanine in the myofibrillar protein pellet by the ratio of ^2^H_5_-phenylalanine/phenylalanine in the free amino acid pool taking into account that ^2^H_5_-phenylalanine was incorporated over a period of 30 min.

### Statistical analysis

Results are expressed as mean ± SEM. Data were analyzed with SigmaStat 3.5 statistical software using a two-way ANOVA followed by the Student-Newman-Keuls *post hoc* testing where appropriate. P<0.05 was considered to be statistically significant.

## Results

### Cancer cachexia and heart weight

Average body mass of the C57BL/6 mice was ∼10 and 26% greater (25.7±1.4 vs. 23.1±1.1 g and 27.3±0.3 vs. 20.1±0.8 g) than that observed in the *Apc^Min/+^* mice at 12 and 20 weeks, respectively. Compared to age-matched controls, heart muscle mass was ∼8 and 6% less (120.4±3.9 vs. 111.2±3.6 mg and 115.8±3.8 vs. 108.8±5.9 mg) in the 12- and 20-week *Apc^Min/+^* mice.

### Decreased protein synthesis rates in the hearts of Apc*^Min/+^* mouse is associated with higher AMPKα and diminished mTOR phosphorylation

**Figure 1:**
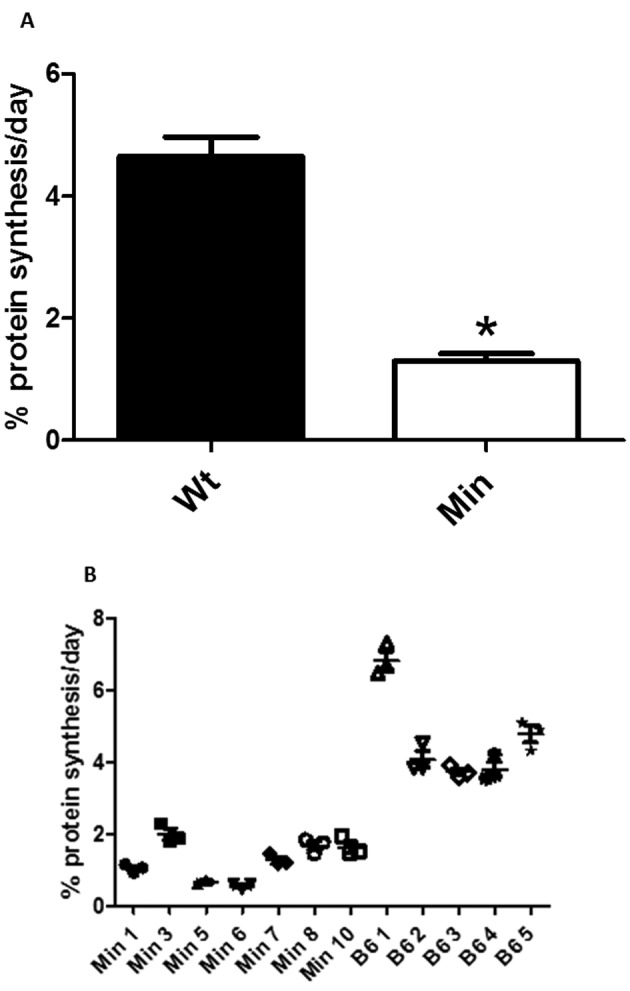
The rate of cardiac myofibrillar protein synthesis is decreased in the cachectic *Apc^Min/+^* mouse. Protein synthesis in mice were determined in whole hearts from 20-week-old BL6 and *Apc^Min/+^* mice by tandem mass spectrometry after intraperitoneal injection of ^2^H_5_-phenylalanine. Percentage protein synthesis/day was calculated from the ratio of radioactivity in the myofibrillar protein pellet to that observed in the free amino acid pool. Mean values ± SEM (A) and raw data (B). An asterisk (^*^) indicates significant differences in *Apc^Min/+^* mice from the BL6 age-matched controls (n=6–7 per group, P<0.05).

**Figure 2:**
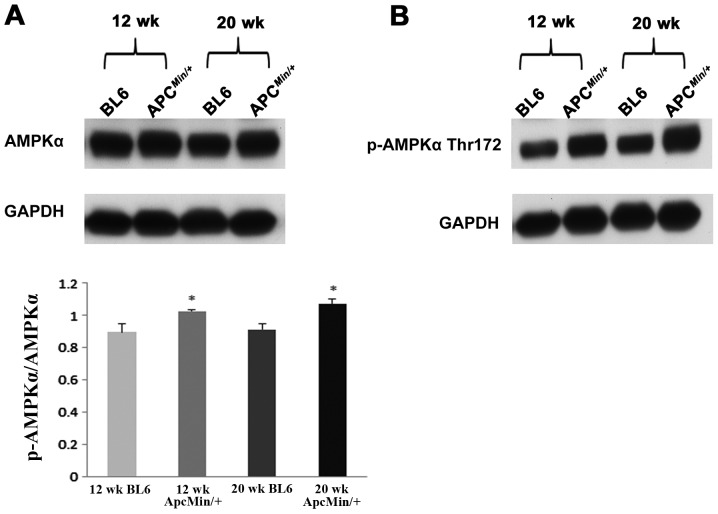
Total and phosphorylated levels of AMPKα are altered in colorectal cancer-induced cachexia. Total and phosphorylated levels of AMPKα at Thr172 in BL6 and *Apc^Min/+^* mice at 12 and 20 weeks. Data are normalized relative to the amount of GAPDH and results are expressed as the ratio of phosphorylated to total levels of AMPKα in arbitrary units for comparison. An asterisk (*) indicates significant differences in *Apc^Min/+^* mice from the BL6 age-matched controls (n=4–6 per group, P<0.05).

**Figure 3:**
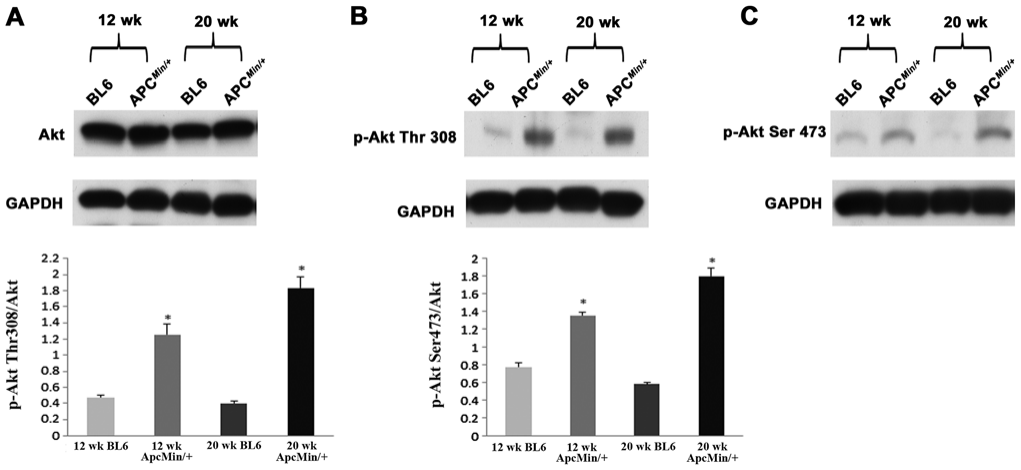
Cancer cachexia is associated with phosphorylation of Akt in the heart. Heart muscles from BL6 and *Apc^Min/+^* were analyzed by western blot analysis for cachexia-related changes in total and phosphorylated levels of Akt at Ser308 and Thr473. Data is normalized relative to the amount of GAPDH and results are expressed as the ratio of phosphorylated to total levels of Akt in arbitrary units for comparison. An asterisk (^*^) indicates significant differences in *Apc^Min/+^* mice from the BL6 age-matched controls (n=4–6, per group P<0.05).

**Figure 4:**
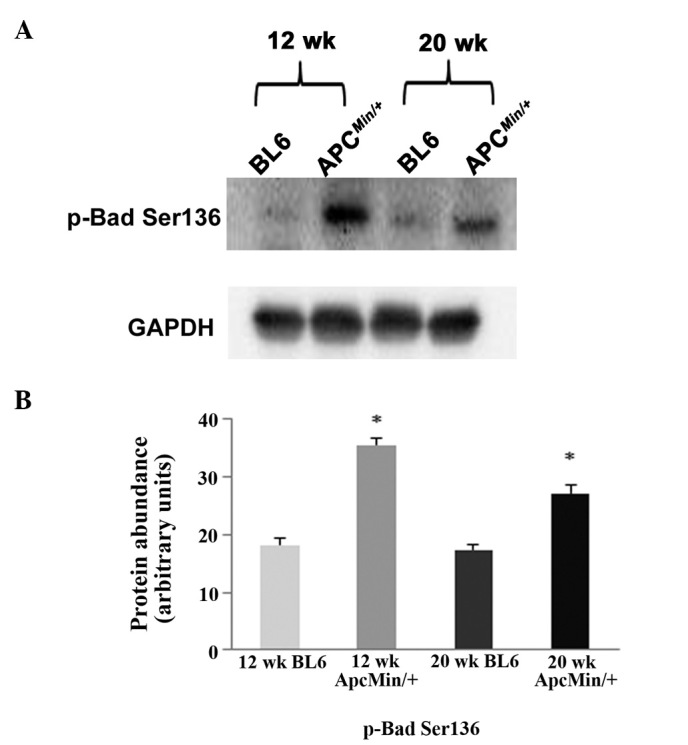
The Akt-dependent phosphorylation of Bad is increased in the hearts of cachexic *Apc^Min/+^* mice. Phosphorylated levels of Bad at Ser136 in BL6 and *Apc^Min/+^* mice at 12 and 20 weeks. Data are normalized relative to the amount of GAPDH and results are expressed as arbitrary units for comparison. An asterisk (*) indicates significant differences in *Apc^Min/+^* mice from the BL6 age-matched controls (P<0.05).

**Figure 5:**
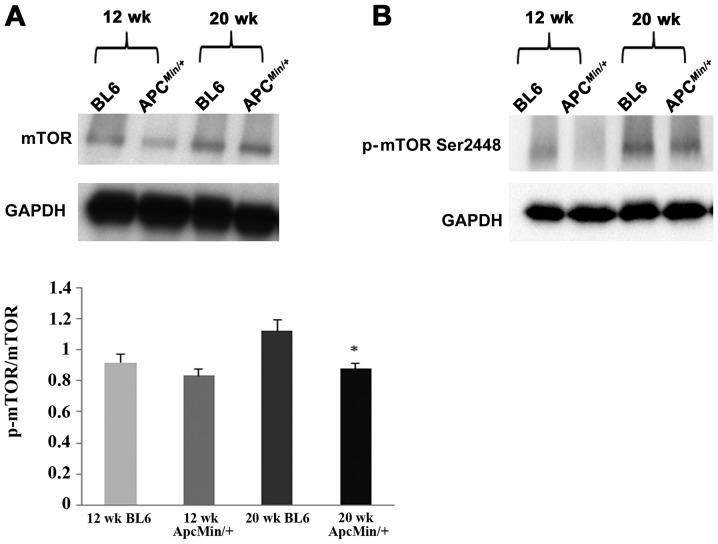
Cancer cachexia decreases the phosphorylation of mTOR in the heart. Total and phosphorylated levels of mTOR at Ser2448 were analyzed by immunoblotting. Data are normalized relative to the amount of GAPDH and results are expressed as the ratio of phosphorylated to total levels of mTOR in arbitrary units for comparison. An asterisk (*) indicates significant differences in *Apc^Min/+^* mice from the BL6 age-matched controls (n=4–6 per group, P<0.05).

**Figure 6:**
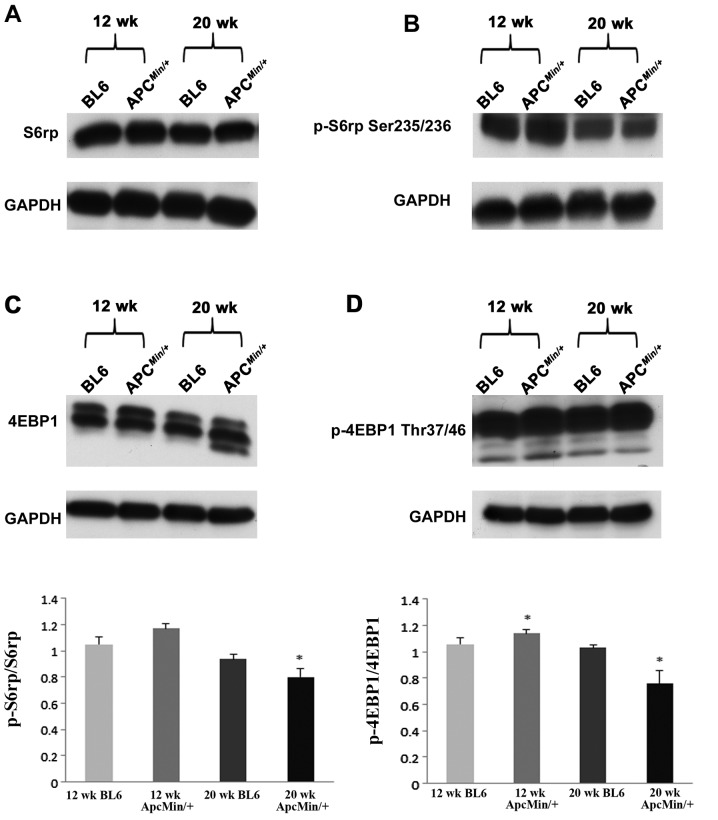
Cancer cachexia decreases the phosphorylation of S6 ribosomal protein and 4EBP1 in the heart. Immunoblot analysis for total and phosphorylated S6rp at Ser235/236 in BL6 and *Apc^Min/+^* mice of 12 and 20 weeks (A and B). Total and phosphorylated levels of 4EBP1 at Thr 37/46 in BL6 and *Apc^Min/+^* mice at 12 and 20 weeks (C and D). Data are normalized relative to the amount of GAPDH and results are expressed as the ratio of phosphorylated to total levels of S6rp and 4EBP1, respectively, in arbitrary units for comparison. An asterisk (*) indicates significant differences in *Apc^Min/+^* mice from the BL6 age-matched controls (n=4–6 per group, P<0.05).

Compared to age-matched C57BL/6 mice, the rate of myofibrillar protein synthesis as determined by tandem mass spectrometry was less in the 20-week-old Apc^Min/+^ mice (Fig. 1). To investigate the effects of cancer cachexia on potential regulators of protein synthesis, we next examined the cardiac lysates for changes in the phosphorylation of AMPKα, Akt and mTOR. Compared to that observed in the control animals, the ratio of phosphorylated to total levels of AMPKα were ∼14 and ∼17% higher in the 12- and 20-week-old *Apc^Min/+^* mice, respectively (P<0.05; Fig. 2). Conversely, there was no difference in Akt protein content among different groups (Fig. 3). Compared to C57BL/6 controls, the phosphorylated to total levels of Akt (Ser308) was ∼2.6- and ∼4.5-fold higher in the 12- and 20-week-old *Apc^Min/+^* mice, respectively (P<0.05; Fig. 3). Similarly, with cancer the amount of phosphorylated to total levels of Akt (Thr473) was ∼1.75- and ∼3.1-fold higher in 12- and 20-week-old *Apc^Min/+^* mice, respectively (P<0.05; Fig. 3). The phosphorylation of Bad (Ser136) was ∼100 and ∼74% higher in the 12-and 20-week-old *Apc^Min/+^* mice, respectively (P<0.05; Fig. 4). Compared to age-matched C57BL/6 mice, the phosphorylated mTOR expression was slightly decreased by ∼9% in 12-week-old *Apc^Min/+^* mice. However, at 20 weeks of age phosphorylated mTOR expression was reduced by ∼21% (P<0.05; Fig. 5), demonstrating a suppression of cardiac mTOR signaling during the progression of cachexia. Similarly, the ratio of phosphorylated to total level of S6rp was ∼14% lower in the hearts of 20-week-old *Apc^Min/+^* mice (P<0.05; Fig. 6). Further analysis revealed that the ratio of phosphorylated to total 4EBP1 was 8% higher in the hearts of 12-week-old *Apc^Min/+^* mice and ∼26% lower in the hearts of 20-week-old *Apc^Min/+^* mice when compared to age-matched controls (P<0.05; Fig. 6).

### Cachexia increases the expression of Beclin1 but does not alter protein ubiquitination or involve the activation of caspase-3

**Figure 7:**
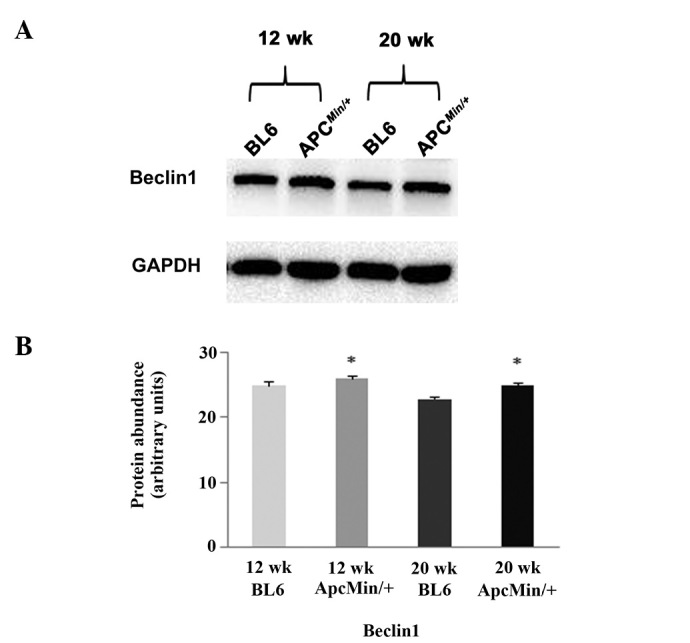
Cardiac atrophy is associated with increased Beclin1 protein expression. Total levels of Beclin1 in BL6 and *Apc^Min/+^* mice at 12 and 20 weeks of age. Data are normalized relative to the amount of GAPDH and results are expressed as arbitrary units for comparison. An asterisk (*) indicates significant differences in *Apc^Min/+^* mice from the BL6 age-matched controls (n=4–6 per group, P<0.05).

It is thought that muscle utilizes three different proteolytic pathways to regulate muscle protein breakdown: calcium-dependent calpains, the ubiquitin-proteasome system and autophagy (13). Compared to that observed in the control animals, no evidence of increased protein ubiquitination, increased caspase-3 activation or apoptosis with cancer was observed (data not shown). Conversely, immunoblot analysis revealed that the expression of the autophagy regulator Beclin1 was ∼5 and ∼10% higher in the hearts of the 12- and 20-week-old *Apc^Min/+^* mice, respectively (P<0.05; Fig. 7).

## Discussion

Cancer cachexia is a life endangering paraneoplastic syndrome that can lead to the loss of muscle mass, decreased body weight, fatigue and generalized weakness that is thought to be responsible for nearly one-third of cancer deaths (13). How cancer may affect cardiac mass is not well understood. Heart weight in the *Apc^Min/+^* mouse colorectal cancer model was decreased by 8 and 6% at 12 and 20 weeks when compared to age-matched control animals. This loss in muscle mass was much smaller than that observed in the gastrocnemius (−41%) and soleus (−26%) in the 20-week-old *Apc^Min/+^* mouse suggesting that muscle atrophy with cachexia may be regulated differently in different muscle types (11). In addition, it is likely that the degree of cardiac muscle loss may differ between cancer models. For example, using the Yoshida tumor implant model, Costelli *et al* demonstrated significant decreases in cardiac mass within six to ten days of implant (14). More recently, Cosper and Leinwand using colon-26 adenocarcinoma implants, showed that cardiac mass was decreased by 8 and 21% at 15 and 27 days post-implantation, respectively (13). The reason why different cancer models may affect the regulation of heart mass differently is not clear but could be related to the amount or type of humoral factors released by the tumor. For example, the muscle wasting seen in the Yoshida hepatoma ascites model is generally considered to be the result of elevations in TNF-α while muscle loss in the colon-26 adenocarcinoma tumor implant model has been shown to be associated with increases in the amount of IL-1, IL-6, IL-12 and TNF-α (15–17). Whether differences in the type or amount of circulating mediators can, by themselves, entirely account for the differences in cardiac muscle loss between models remains to be determined.

In the case of the *Apc^Min/+^* mouse model, it is generally thought that the wasting induced in this model can be explained, at least in part, due to an elevations in serum IL-6 levels (3,11,18). How IL-6 may affect cardiac structure or function is poorly understood. Like that seen in the *Apc^Min/+^* mouse, recent data have suggested that burn injury and sepsis are also characterized by increases in IL-6 levels which, at least in these models, is associated with cardiac inflammation, increased apoptosis, and depressed contractile function (19,20). Others have demonstrated that increased IL-6 levels are associated with diminished muscle protein synthesis (21) and AMPK activation (22). Our findings of diminished cardiac protein synthesis rates (Fig. 1) and evidence of increased AMPKα phosphorylation in both the 12- and 20-week-old *Apc^Min/+^* mice support this contention (Fig. 2). AMPK activation can act as a molecular switch that turns on energy conserving pathways while shutting down the energy consuming pathways such as protein synthesis (23). Whether the increase in AMPKα we observed is due to elevations in IL-6, changes in nutrient uptake secondary to colon tumor load, or some combination of the two is currently unclear.

In an effort to extend our understanding of how cachexia might affect translational signaling in the *Apc^Min/+^* mouse, we next examined whether changes in cardiac mass were associated with differences in the phosphorylation of Akt and mTOR. Akt is a serine/threonine kinase which is involved in the regulation of cell metabolism and cell death (24). With cancer, we found an elevation in Akt phosphorylation at both Thr308 and Ser473 residues which is consistent with notion of Akt activation (Fig. 3). To explore whether the observed Akt activation with cachexia might be anti-apoptotic in nature, we examined the effect of cachexia on the phosphorylation of Bad. Like Akt, Bad is intimately involved in regulating the transition between cellular life and death. In its unphosphorylated form Bad is strongly apoptotic (25,26). Conversely, when phosphorylated at Ser136 by Akt, the apoptotic activity of Bad is inhibited and cell death is prohibited. Compared to that seen in the control animals, the phosphorylation of Bad (Ser136) was significantly higher in the *Apc^Min/+^* mouse hearts (Fig. 4). Given that diminished Akt signaling may also be involved in increasing proteasome activity during skeletal muscle atrophy (27), we next examined if cardiac muscle loss was associated with increases in protein ubiquitination. As expected from our findings of increased Akt phosphorylation and similar to that others have seen investigating cardiac atrophy in the C-26 adenocarcinoma tumor implant model (13) we did not find any evidence of increased protein ubiquitination or cardiac apoptosis (data not shown). Taken together, these data suggest that the activation of Akt in the cachectic heart may be a compensatory response initiated to minimize the apoptotic effects of circulating humoral factors, e.g., IL-6 and TNF-α, to preserve cardiac mass and function.

Similar to Akt, the activation of mTOR is required for cell survival (28). In addition to its effects on cellular survival, the activation (phosphorylation) of mTOR and its substrates are required for the induction of protein synthesis (29). Unlike that observed by us for Akt, we found significant decrease in the ratio of phosphorylated to total mTOR with cachexia (Fig. 5). To confirm these findings, we next examined the phosphorylation of downstream mTOR substrates, S6rp and 4EBP1. As expected from our assessment of mTOR phosphorylation and our findings of diminished protein synthesis rates, we observed that the amount of phosphorylated (active) S6rp and 4EBP1 were both diminished with cachexia (Fig. 6). This finding is consistent with the recent study of White and colleagues who demonstrated diminished myofibrillar synthesis rates in the skeletal muscles of the *Apc^Min/+^* mouse at similar time-points (10).

Autophagy is a mechanism for cell protection in which intracellular substances are degraded and the subsequent products are recycled again (30). On the basis of recent data suggesting that cancer cachexia is associated with increased autophagy (13) and other findings demonstrating that increased AMPK phosphorylation can induce the expression of the autophagy proteins (7) we next examined if cachexia in the *Apc^Min/+^* mice was associated with increases in autophagy protein Beclin1. Consistent with our findings of decreased cardiac mass, we observed that cancer cachexia in the *Apc^Min/+^* mice was associated with slightly elevated levels of Beclin1 (Fig. 7). These findings suggest that changes in cardiac mass in the *Apc^Min/+^* mouse may be associated with autophagy.

**Figure 8:**
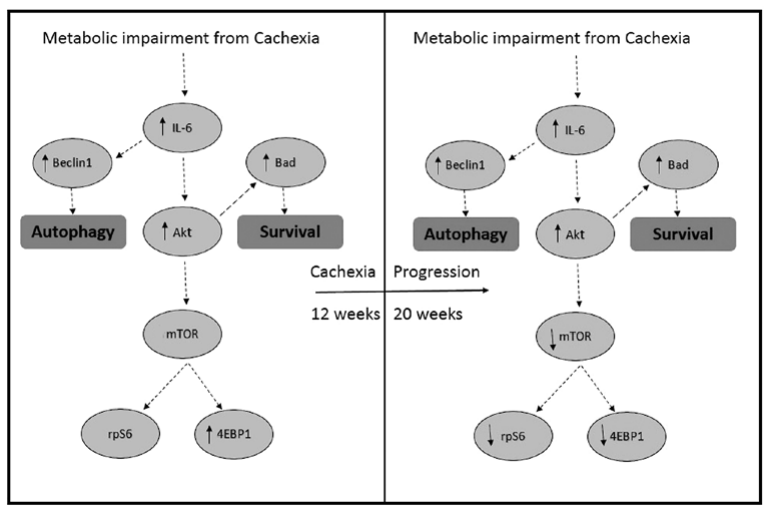
Schematic representation of different pathways involved in cancer-induced cardiac cachexia in 12- and 20-week-old *Apc^Min/+^* mice. Recent studies along with the data obtained in the present study suggest that cancer cachexia is characterized by gradual increase in protein degradation and decrease in protein synthesis with age. Solid arrows correspond to increase or decrease in activation of the respective protein in the 12- and 20-week-old *Apc^Min/+^* mice compared with age-matched BL6 controls.

Taken together, our data demonstrate that cachexia in the *Apc^Min/+^* mouse model is associated with a mild form of cardiac atrophy that may be caused by decreases in the rate of myofibrillar protein synthesis and the suppression of anabolic signaling through mTOR. We hypothesize that this decrease in protein synthesis is accompanied by increases in cardiac autophagy as suggested by our findings of increased AMPK phosphorylation and Beclin1 protein expression. We suspect that the activation of Akt and Bad observed in the present study are more related to efforts directed at maintaining cell survival rather than increasing protein synthesis (Fig. 8). Future studies examining tumor humoral factors or mediators of systemic inflammation will no doubt be useful in furthering our understanding of how colorectal cancer may affect cardiac structure and function.
